# Immunoglobulin M seroneutralization for improved confirmation of Japanese encephalitis virus infection in a flavivirus-endemic area

**DOI:** 10.1093/trstmh/trac036

**Published:** 2022-05-18

**Authors:** Tehmina Bharucha, Nazli Ayhan, Boris Pastorino, Sayaphet Rattanavong, Manivanh Vongsouvath, Mayfong Mayxay, Anisone Changthongthip, Onanong Sengvilaipaseuth, Ooyanong Phonemixay, Jean-David Pommier, Christopher Gorman, Nicole Zitzmann, Paul N Newton, Xavier de Lamballerie, Audrey Dubot-Pérès

**Affiliations:** Department of Biochemistry, University of Oxford, Oxford, UK; Lao-Oxford-Mahosot Hospital-Wellcome Trust-Research Unit, Microbiology Laboratory, Mahosot Hospital, Vientiane, Lao PDR; Unité des Virus Émergents, Aix-Marseille Univ-IRD 190-Inserm 1207, Marseille, France; Unité des Virus Émergents, Aix-Marseille Univ-IRD 190-Inserm 1207, Marseille, France; Lao-Oxford-Mahosot Hospital-Wellcome Trust-Research Unit, Microbiology Laboratory, Mahosot Hospital, Vientiane, Lao PDR; Lao-Oxford-Mahosot Hospital-Wellcome Trust-Research Unit, Microbiology Laboratory, Mahosot Hospital, Vientiane, Lao PDR; Lao-Oxford-Mahosot Hospital-Wellcome Trust-Research Unit, Microbiology Laboratory, Mahosot Hospital, Vientiane, Lao PDR; Institute of Research and Education Development, University of Health Sciences, Ministry of Health, Vientiane, Lao PDR; Centre for Tropical Medicine and Global Health, Nuffield Department of Medicine, University of Oxford, Oxford, UK; Lao-Oxford-Mahosot Hospital-Wellcome Trust-Research Unit, Microbiology Laboratory, Mahosot Hospital, Vientiane, Lao PDR; Lao-Oxford-Mahosot Hospital-Wellcome Trust-Research Unit, Microbiology Laboratory, Mahosot Hospital, Vientiane, Lao PDR; Lao-Oxford-Mahosot Hospital-Wellcome Trust-Research Unit, Microbiology Laboratory, Mahosot Hospital, Vientiane, Lao PDR; Epidemiology and Public Health Unit, Institut Pasteur du Cambodge, Phnom Penh, Cambodia; Institut Pasteur, Biology of Infection Unit, Paris, France; Inserm U1117, Paris, France; Intensive Care Department, University Hospital of Guadeloupe, France; Virology Unit, Institut Pasteur du Cambodge, Phnom Penh, Cambodia; Department of Biochemistry, University of Oxford, Oxford, UK; Lao-Oxford-Mahosot Hospital-Wellcome Trust-Research Unit, Microbiology Laboratory, Mahosot Hospital, Vientiane, Lao PDR; Centre for Tropical Medicine and Global Health, Nuffield Department of Medicine, University of Oxford, Oxford, UK; Unité des Virus Émergents, Aix-Marseille Univ-IRD 190-Inserm 1207, Marseille, France; Lao-Oxford-Mahosot Hospital-Wellcome Trust-Research Unit, Microbiology Laboratory, Mahosot Hospital, Vientiane, Lao PDR; Unité des Virus Émergents, Aix-Marseille Univ-IRD 190-Inserm 1207, Marseille, France; Centre for Tropical Medicine and Global Health, Nuffield Department of Medicine, University of Oxford, Oxford, UK

**Keywords:** diagnostics, flavivirus, Laos, neglected tropical disease, neurological infection, seroneutralization

## Abstract

**Background:**

The mainstay of diagnostic confirmation of acute Japanese encephalitis (JE) involves detection of anti-JE virus (JEV) immunoglobulin M (IgM) by enzyme-linked immunosorbent assay (ELISA). Limitations in the specificity of this test are increasingly apparent with the introduction of JEV vaccinations and the endemicity of other cross-reactive flaviviruses. Virus neutralization testing (VNT) is considered the gold standard, but it is challenging to implement and interpret. We performed a pilot study to assess IgG depletion prior to VNT for detection of anti-JEV IgM neutralizing antibodies (IgM-VNT) as compared with standard VNT.

**Methods:**

We evaluated IgM-VNT in paired sera from anti-JEV IgM ELISA-positive patients (JE n=35) and negative controls of healthy flavivirus-naïve (n=10) as well as confirmed dengue (n=12) and Zika virus (n=4) patient sera. IgM-VNT was subsequently performed on single sera from additional JE patients (n=76).

**Results:**

Anti-JEV IgG was detectable in admission serum of 58% of JE patients. The positive, negative and overall percentage agreement of IgM-VNT as compared with standard VNT was 100%. A total of 12/14 (86%) patient samples were unclassified by VNT and, with sufficient sample available for IgG depletion and IgG ELISA confirming depletion, were classified by IgM-VNT. IgM-VNT enabled JE case classification in 72/76 (95%) patients for whom only a single sample was available.

**Conclusions:**

The novel approach has been readily adapted for high-throughput testing of single patient samples and it holds promise for incorporation into algorithms for use in reference centres.

## Introduction

Progress has been made in the implementation of vaccination programmes for Japanese encephalitis virus (JEV) in endemic areas.^1–3^ Nonetheless, gaps remain in understanding the epidemiology of the disease.^2,4^ Incorporation of JEV immunization in routine schedules and coverage remain suboptimal and there is inadequate surveillance to identify vaccine failure and JEV geographical expansion.^2,5–8^

Detection of JEV nucleic acid is highly specific and provides additional molecular information.^7,9^ However, viraemia is brief and low in humans and JEV RNA is rarely detected.^10^ Correspondingly, serological methods are the mainstay of diagnostic confirmation. The World Health Organization (WHO)-recommended test is the anti-JEV immunoglobulin M (IgM) capture enzyme-linked immunosorbent assay (JEV MAC-ELISA) to be performed and interpreted alongside an anti-dengue virus (DENV) MAC-ELISA.^11^ The availability of commercial kits has facilitated widespread use of the JEV MAC-ELISA as the standard test. However, in line with other flaviviruses, there are increasingly recognized problems with specificity.^12–15^ For this reason, the Centers for Disease Control and Prevention (CDC) recommends that positive results obtained through JEV MAC-ELISA undergo confirmation by neutralizing antibody (NAb) testing.^16^

Gold-standard serological confirmation of JEV infection involves assessment of NAb titres using a virus neutralization test (VNT). This is more specific^13,17^ than the JEV MAC-ELISA. Conventional VNT methods involve a plaque reduction neutralisation test (PRNT), however, laboratories are increasingly adopting high-throughput 96-well formats with comparable results.^18^ The high VNT requirements limit implementation: testing involves relatively large (>150 µL) sample volumes, the need for paired samples, biosafety 3 category laboratories, reference virus and cell strains and technical expertise. Indeed, interpreting VNT results is challenging due to cross-reactivity that is attributable to anamnestic responses related to immunological reactions against a previously encountered flavivirus.^19^ As there are specific major overlaps in the distribution of JEV and other flaviviruses, contemporaneous VNT for other endemic flaviviruses is required. In Asia, this involves testing for DENV serotypes 1–4, Zika virus (ZIKV) and, in some areas, West Nile virus (WNV).^20^ All of these viruses can manifest as neurological complications.^21^

Multiple methods have been attempted to mitigate cross-reactivity and anamnestic response interference in serological testing for non-JEV flaviviruses. These include analysis of IgA,^22–31^ IgG subclasses,^25^ antibody avidity,^22,32–35^ incorporation of blocking agents^34,36^ and production of specific monoclonal antibodies for identification of specific viral epitopes.^37–41^ A modification of VNT, involving prior depletion of IgG, has been successfully performed for ZIKV^19^ and DENV infections.^42,43^ The underlying principle is that long-lasting IgG responses from vaccination and previous infection are major contributors to non-specific VNT results. IgG removal results in detection of specific neutralizing IgM antibodies, which are markers of acute infection.

We performed a pilot study to evaluate the utility of IgG depletion prior to VNT (IgM-VNT) to detect anti-JEV IgM neutralizing antibody for confirming acute JEV infection.

## Methods

### Patient samples

A prospective study of central nervous system (CNS) infections has been conducted at Mahosot Hospital, Vientiane, Laos, since 2003. Methods and results from 2003 to 2011 have been described.^44^ Patients from 2014 to 2017 were included in the Southeast Asia Encephalitis Project.^45^ The laboratory also receives samples from patients from other hospitals around Vientiane City (i.e. Friendship, Children's and Setthathirat Hospitals). Written informed consent was obtained from patients or responsible guardians. Anti-JEV and anti-DENV IgM were detected by the Japanese encephalitis/dengue IgM combo ELISA (Panbio, Brisbane, QLD, Australia; now Alere) until July 2014, for which result interpretation included a ratio between DENV and JEV. After August 2014, as per WHO recommendations, the JEV IgM ELISA (Inbios, Seattle, WA, USA) was utilized. All samples used were aliquoted and stored at −80°C. This pilot study involved a convenience sample of consecutive patients with available specimens to be tested; hence a sample size calculation was not performed.

Suspected JE patients included in this study had anti-JEV IgM detected by MAC-ELISA in cerebrospinal fluid (CSF) or seroconversion between acute and follow-up serum, no other pathogen detected in any body fluid and a sufficient volume of acute and/or follow-up serum for VNT. Patients with DENV and JEV RNA or DENV non-structural protein 1 (NS1) in serum or CSF were excluded.

Negative controls included samples from three groups: healthy flavivirus-naïve blood donors living in Puy-de-Dôme, in central France; ZIKV VNT-confirmed sera collected in Peru in the framework of a seroprevalence study^46^; and DENV infection patients from the Laos CNS study (study details reported in the section on suspected JE patients above), confirmed by IgM and/or NS1 ELISA and negative for anti-JEV IgM. All procedures relating to the conduct, evaluation and documentation of the study have been conceived in agreement with the good clinical practices and ethical principles of the Helsinki Declaration. Written informed consent was obtained from all subjects included in the study. All data and samples were anonymised.

### Anti-JEV IgG ELISA

Anti-JEV IgG was detected using the Euroimmun ELISA kit (Lübeck, Germany) according to manufacturer's instructions. A standard curve using three calibration samples was used to calculate the concentration of antibodies in relative units (RU)/mL for each sample using optical density results; <16 RU/mL was negative, ≥16–<22 RU/mL was equivocal and ≥22 RU/mL was positive.

### IgG depletion

IgG depletion was performed using Protein G HP SpinTrap/Ab Spin Trap columns (28-4083-47; Cytiva, Marlborough, MA, USA). These contain recombinant protein G, a protein present in group G *Streptococcus* with high affinity for IgG. An in-house method developed by the French National Centre for Arboviruses was used, substituting commercial binding buffer by phosphate-buffered saline (PBS). Two IgG depletion columns were used for 100–150 µL sample serum. Columns were inverted three times and briefly vortexed. Each column was inserted in a 2-mL tube and centrifuged. All centrifugation steps were performed at 500 *g* for 2 min. The subsequent eluate was discarded, 600 µL of PBS added to each column and centrifuged again. Columns were transferred to clean 2-mL tubes and 100–150 µL of sample was added to one column and incubated at room temperature for 4 min before centrifugation. The eluate was transferred to the second column, incubated at room temperature for 4 min and centrifuged again. The final eluate was stored at −20°C until the VNT.

### VNT

Two-fold dilutions from 1/20 to 1/2560 of each serum sample were tested in duplicate by VNT for JEV, DENV1–4, ZIKV and WNV. Serum dilutions from 1/10 to 1/1280 were prepared and mixed in a 1:1 ratio with 100 TCID50 viral suspension (Table 1) using epMotion 5075 (Eppendorf, Hamburg, Germany) in a 96-well microplate (Figure S1). Negative controls containing minimum essential medium (MEM), with or without serum, were included in each microplate. Plates were incubated at 37°C for 2 h. A 100-µL suspension of Vero cells (ATCC CCL-81) containing approximately 2×10^5^ cells/mL, was added to each well using the epMotion 5070 (Eppendorf) and incubated at 37°C in a 5% carbon dioxide incubator. After 5–7 d, microplates were read under an inverted microscope. Two investigators read the results for each replicate to identify the end dilution at which there was no cytopathic effect, with a third investigator to resolve disagreement. For duplicates, the geometric mean of end dilutions was calculated and reported as an NAb titre and ≥40 was considered as positive.^47,48^ Suspected JE patients were categorized as acute JE positive, confirmed or compatible, JE negative and unknown, according to the criteria in Figure 2.

**Table 1.** Virus strain used in VNTs

Virus	Strain	Country of isolation	GenBank number	EVAg number	Titre (TCID_50_/mL)	Day read
JEV	Laos 2009	Laos	KC196115	001V-02217	2×10^9^	5
WNV	UVE/WNV/2008/US/R94224	USA	–	001V-02224	2.1×10^7^	5
ZIKV	ZIKV strain H/PF/2013 French Polynesia	French Polynesia	KJ776791	–	3.7×10^6^	5
DENV-1	DENV1 2012	Saint Vincent and the Grenadines	VC16692	001V-02335	3.1×10^7^	7
DENV-2	UVE/DENV-2/1998/MQ/703	Martinique	AF208496	–	6.7×10^4^	5
DENV-3	UVE/DENV-3/2001/MQ/2023	Martinique	AH011666	–	4.5×10^5^	6
DENV-4	UVE/DENV-4/1998/ID/814	Indonesia	–	–	3×10^6^	6

EVAg: European Virus Archive – GLOBAL; TCID_50_: 50% tissue culture infective dose.

## Results

**Figure 1.**
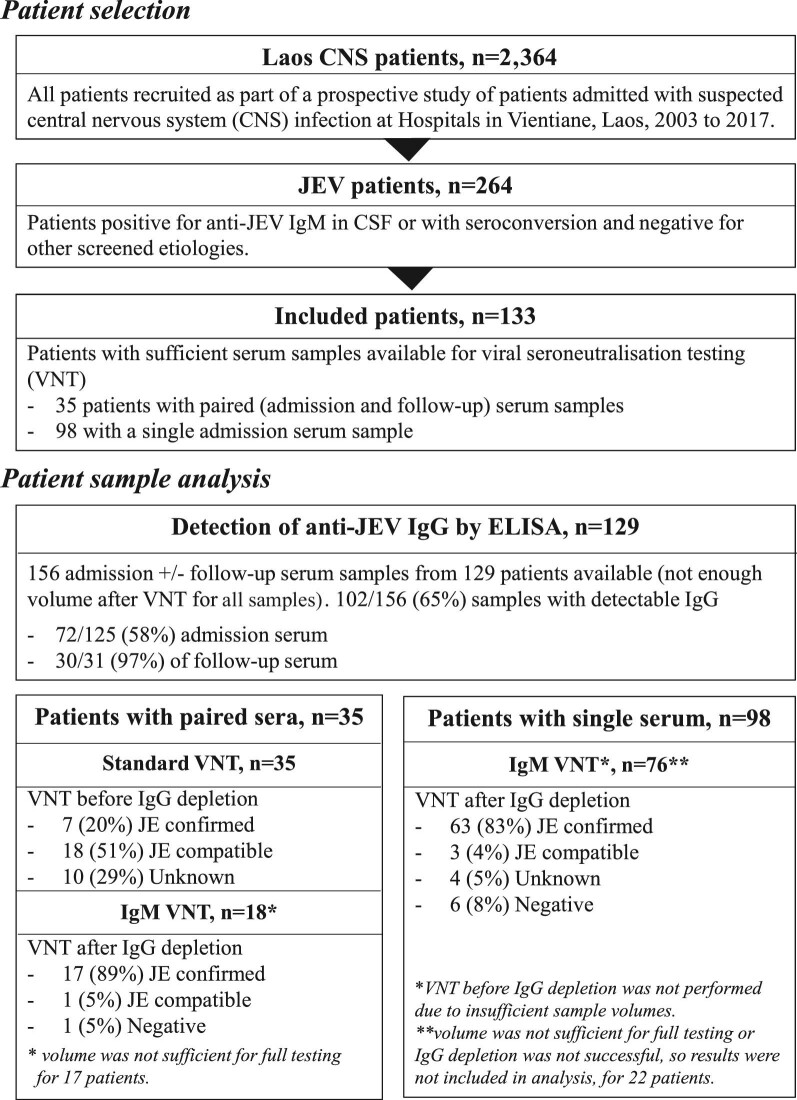
Summary of the suspected JE patient samples tested.

From 2003 to March 2021, 264 patients with suspected CNS infection were positive for anti-JEV IgM (in CSF or with seroconversion) and negative for other screened aetiologies^44^ (see Figure 1). Paired serum samples (admission and follow-up) were available for 35 patients and a single acute sample for 98 patients. Among these 133 included patients, 130 (98%) had anti-JEV IgM detected in CSF and 3 (2%) demonstrated IgM seroconversion only (no anti-JEV IgM in CSF) in paired sera. The median age of the patients was 11 y (interquartile range [IQR] 6–20) and 32% (43/133) were female. The median duration of illness on admission was 5 d (IQR 4–6) and the median time between admission and follow-up serum collection was 14 d (IQR 10–25).

**Figure 2.**
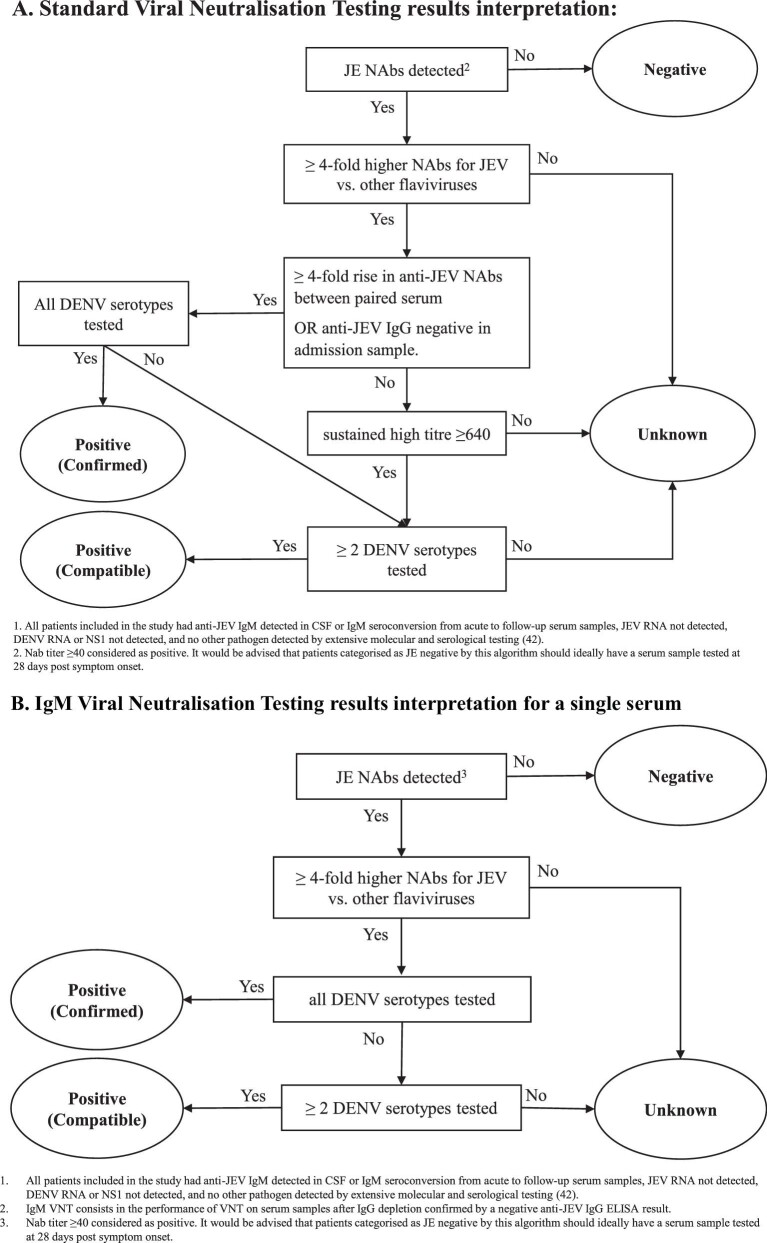
Criteria for interpretation of the results and patient categorisation for JE status.

### IgG depletion

A total of 102/156 (65%) serum samples, including 72/125 (58%) admission sera and 30/31 (97%) follow-up sera, were anti-JEV IgG positive by ELISA before IgG depletion. Seventy samples had sufficient volumes to be tested for anti-JEV IgG by ELISA after IgG depletion. Fifty-nine (84%) were negative or equivocal after IgG depletion. Six samples were equivocal before IgG depletion and all of these were negative after IgG depletion. Samples that remained positive after IgG depletion demonstrated decrease in the titre, however, the starting anti-JEV IgG result in these cases was high, all >125 RU/mL (positive >22 RU).

### VNT for the patients with paired serum samples

VNT results prior to IgG depletion enabled classification of 25/35 (71%) patients as JE positive, 7 (20%) confirmed, 18 (51%) compatible; and 10 (29%) as unknown (Table 2 and Table S2). Eighteen of these patients had sufficient serum available for IgM-VNT in at least one sample. The results enabled reclassification through the removal of cross-reactive IgG to other viruses and the specific detection of anti-JEV IgM, such that 17 (94%) were classified as JE positive, 16 (89%) confirmed, 1 (6%) compatible; and 1 (6%) as JE negative. Five patients classified as unknown by VNT did not have sufficient acute and/or follow-up sample to perform IgG depletion and/or anti-JEV IgG ELISA testing.

**Table 2.** VNT antibody titre in acute and follow-up serum samples for patients with positive anti-JEV IgM capture ELISA

			Before IgG depletion (standard VNT)									After IgG depletion (IgM-VNT)								
					NAb titre									NAb titre						
Patient number	Sample type	Days of illness	Class	JEV IgG	JEV	D1	D2	D3	D4	ZIK	WN	Class	JEV IgG	JEV	D1	D2	D3	D4	ZIK	WN
1597	Adm	5	Conf	−	1280	Neg	Neg	Neg	Neg			Conf	−^a^	160^b^	Neg^b^	Neg^b^	Neg^b^	Neg^b^		Neg^b^
	FU	59		+	2560	14	14	Neg	20					640^b^	Neg^b^	Neg^b^	Neg^b^	Neg^b^		
1704	Adm	5	Conf	+	640	20	Neg	20	28			Conf	−	640^b^	Neg^b^	Neg^b^	Neg^b^	Neg^b^	Neg^b^	Neg^b^
	FU	13		+	2560	40	Neg	28	80					2560 ^b^	20^b^	Neg^b^	Neg^b^	Neg^b^	Neg^b^	Neg^b^
829	Adm	4	Conf	−	1280	Neg	Neg	Neg	Neg	Neg	14	Conf	−	640^b^	Neg^b^	Neg^b^	Neg^b^	Neg^b^	Neg^b^	Neg^b^
	FU	21		+	2560	Neg	Neg	Neg	14	Neg^b^										
908	Adm	4	Conf	+	40	160	160	40	113	Neg		Conf								
	FU	14		+	2560	20	Neg	Neg	14				−	2560	Neg^b^	Neg^b^	Neg^b^	Neg^b^	Neg^b^	
928	Adm	5	Conf	−	1810	20	Neg	Neg	Neg	Neg	56	Conf	−	2560 ^b^	Neg^b^	Neg^b^	Neg^b^	Neg^b^	Neg^b^	Neg^b^
	FU	44		+	2560	Neg	Neg	Neg	14				−	2560	Neg^b^	Neg^b^	Neg^b^	Neg^b^	Neg^b^	Neg^b^
2078	Adm	7	Conf	Eq	160	20	Neg	Neg	Neg			Conf	−							
	FU	17		+	2560	Neg	Neg	20	20				−	≥2560	Neg	Neg	Neg	Neg		
101	Adm	4	Conf	−	2560	Neg	Neg	Neg	Neg			Conf	−	453	Neg	Neg	Neg	Neg		
	FU	6		−	2560	Neg	Neg	40	Neg				−	≥2560	Neg	Neg	Neg	Neg		
																				
1610	Adm	6	Comp	+	2560	14	Neg	Neg	20			Conf	−	1280^b^	Neg^b^	Neg^b^	Neg^b^	Neg^b^	Neg^b^	Neg^b^
	FU	40		+	2560	160	20	Neg	113					2560	Neg	Neg	Neg	Neg	Neg	Neg
483	Adm	7	Comp	+	2560	Neg	Neg	Neg	Neg			Conf	−	2560 ^b^	Neg^b^	Neg^b^	Neg^b^	Neg^b^	Neg^b^	Neg^b^
	FU	21		+	2560	20	20	Neg	20	Neg	Neg			2560 ^b^	Neg^b^	Neg^b^	Neg^b^	Neg^b^	Neg^b^	Neg^b^
884	Adm	5	Comp	+	2560	40	Neg	Neg	40	Neg	40	Conf	−	1280^b^	Neg^b^	Neg^b^	Neg^b^	Neg^b^	Neg^b^	Neg^b^
	FU	19		+	2560	Neg	Neg	Neg	20				−	2560 ^b^	Neg^b^	Neg^b^	Neg^b^	Neg^b^	Neg^b^	Neg^b^
1074	Adm	6	Comp	Eq	452		Neg^b^	Neg	Neg			Conf								
	FU	84		+	1810	40	40	Neg	80				−	1280^b^	Neg^b^	Neg^b^	Neg^b^	Neg^b^		Neg^b^
1180	Adm	7	Comp	+	1280	452	640	452	160	Neg		Conf	−	1280^b^	Neg^b^	Neg^b^	Neg^b^	Neg^b^		Neg^b^
	FU	13		+	2560	226	226	160	160	20	80									
2053	Adm	5	Comp	+	2560	20	Neg	Neg	Neg			Conf	−	2560	Neg	Neg	Neg	Neg		
	FU	18		+	1280	80	Neg	40	Neg				−							
775	Adm	3	Comp	−	320	Neg	Neg		Neg			Comp	−^a^	113	Neg	Neg	Neg			
	FU	12		+	640	Neg	Neg		40					640	Neg	Neg	Neg			
																				
5149	Adm	1	Unkn	+	2560	2560	2560	2560	2560			Conf		1280^b^	20^b^	20^b^	20^b^	Neg^b^		
	FU	28		+	2560	2560	2560	2560	2560				−	2560 ^b^	40^b^	20^b^	40^b^	20^b^		Neg^b^
1056	Adm	3	Unkn	+	320	1810	320	320	320	28	40	Conf	−	640^b^	20^b^	Neg^b^	Neg^b^	Neg^b^		Neg^b^
	FU	28		+	2560															
1917	Adm	7	Unkn	+	2560	2560	2560	Neg	453			Conf	−	2560	Neg	Neg	Neg	Neg		
	FU	12		+	1280	2560	2560	Neg	320				−	1280	Neg	Neg	Neg	Neg		
1036	Adm	4	Unkn	+	80	2560	226	320	226	Neg	20	Neg	−	Neg^b^	80^b^	Neg^b^	20^b^	Neg^b^		Neg^b^
	FU	16		+	640	2560 ^b^	1280	2560												

Adm: serum on admission; FU: serum at follow-up; NAb: NAb assessed by VNT, geometric mean calculated from duplicate results, = indeterminate, NAb titre underlined to indicate the maximum dilution tested, neg: no NAb detected in duplicate samples (observation of cytopathic effect) for all serum dilutions tested (lowest = 20); NAb titre ≥40 considered as positive; D1–4: dengue virus 1–4; ZIK: Zika virus; WN: West Nile virus; class: classification for JE status according to criteria in Table 2; Conf: confirmed; Comp: compatible; Unkn: unknown; JEV IgG: anti-JEG IgG detection by ELISA (Euroimmun); +: positive; Eq: equivocal; −: negative.

^a^JEV IgG negative before depletion.

^b^Only one replicate tested or interpretable, the other samples were tested in duplicate.

For the subset of 32 patients classified as JE positive, confirmed or compatible (before or after depletion), the median duration of onset of illness was 5 d (IQR 4–7) and the median duration between paired serum samples was 14 d (IQR 11–24). A total of 17/24 (71%) of these patients had detectable anti-JEV IgG in the admission serum before IgG depletion and 23/24 [96%] had detectable anti-JEV IgG in the follow-up sample.

### Negative control sera

IgM-VNT was performed on three other groups of negative control sera to assess the specificity of the novel method. JEV NAb was not detected by IgM-VNT or VNT in the healthy flavivirus-naïve blood donors (n=10) or ZIKV infection sera (n=4) (see Table S3). In the DENV patient sera, 2/12 (17%) did not have detectable JEV NAb, and for both of these patients, IgM-VNT was performed and was also negative. In the 10/12 (83%) patients with DENV infection with JEV NAb detected by VNT, 8/10 (80%) did not have detectable JEV NAb after IgG depletion. For the remaining two, one did not have a result for IgM-VNT and the other showed negative JEV VNT for admission serum and a low JEV NAb titre of 40 in follow-up serum. There were not sufficient sample volumes available to perform DENV VNT.

### Positive, negative and overall percentage agreement

The IgM-VNT was compared with the reference standard VNT. This was based on results for patients classified as JE positive or negative by standard VNT and with sufficient sera to complete IgM-VNT, i.e. VNT performed after IgG depletion and IgG ELISA to confirm IgG depletion. This included 14 JE-positive and 16 JE-negative patients. Positive, negative and overall percentage agreements (PPA, NPA and OPA, respectively) were all 100% (see Table 3).

**Table 3.** 2×2 table of the results of IgM-VNT as compared with standard VNT

	Reference test (standard VNT)		
IgM-VNT	JE positive^a^, n	JE negative^a^, n	Total, n
JE positive^a^	14	0	14
JE negative^a^	0	16	16
Total	14	16	30

^a^The classification of patients followed the criteria set out in Figure 2.

### VNT after IgG depletion for patients with single acute serum

A total of 76/98 (78%) patient samples had sufficient volumes for IgG depletion, confirmatory IgG ELISA testing and IgM-VNT. Results allowed classification for 72/76 (95%) patients: 70 (92%) JE, 63 (83%) confirmed and 3 (4%) compatible, and 6 (8%) negative. Four (5%) were unknown (Table 4).

**Table 4.** VNT antibody titre for patients with only a single acute serum sample

				After IgG depletion (IgM-VNT)							
					NAb titre						
Patient number	Days of illness	Class	Before IgG depletion, JEV IgG	JEV IgG	JEV	D1	D2	D3	D4	ZIK	WN
34		Conf	−	−	160	Neg	Neg	Neg	Neg	Neg	Neg
37	3	Conf	−	−	640	Neg	Neg	Neg	Neg	Neg	Neg
38	2	Conf	−	−	57	Neg	Neg	Neg	Neg	Neg	
40	14	Conf	−	−	80	Neg	Neg	Neg	Neg	Neg	Neg^a^
44	4	Conf	−	−	57	Neg	Neg	Neg	Neg	Neg	Neg
47	4	Conf	−	−	320	Neg	Neg	Neg	Neg	Neg	Neg
52	4	Conf	−	−	80	Neg	Neg	Neg	Neg	Neg	Neg
53	4	Conf	−	−	57	Neg	Neg	Neg	Neg	Neg	Neg
59	4	Conf	−	−	320	Neg	Neg	Neg	Neg	Neg	Neg
60	1	Conf	−	−	160	Neg	Neg	Neg	Neg	Neg	Neg
64	3	Conf	−	−	80	Neg	Neg	Neg	Neg	Neg	Neg
57	6	Conf	−	−	640	Neg	Neg	Neg	Neg	Neg	Neg
66	8	Conf	−	−	40	Neg	Neg	Neg	Neg	Neg	Neg
73	5	Conf	−	−	1810	Neg	Neg	Neg	Neg	Neg	Neg
76	5	Conf	−	−	320	Neg	Neg	Neg	Neg	Neg	Neg
87	4	Conf	−	−	57	Neg	Neg	Neg	Neg	Neg	Neg
88	6	Conf	−	−	160	Neg	Neg	Neg	Neg	Neg	Neg
92	3	Conf	−	−	905	Neg	Neg	Neg	Neg	Neg	Neg
98		Conf	−	−	320	Neg	Neg	14	Neg	Neg	Neg
101		Conf	−	−	1280	Neg	Neg	Neg	Neg	Neg	Neg
102		Conf	−	−	640	Neg	Neg	28	Neg	Neg	Neg
103		Conf	−	−	320	Neg	Neg	Neg	Neg	Neg	Neg
104		Conf	−	−	226	Neg	Neg	Neg	Neg	Neg	Neg
105		Conf	−	−	160	Neg	Neg	Neg	14	Neg	Neg
111		Conf	−	−	452	Neg	Neg	Neg	Neg	Neg	Neg
112		Conf	−	−	160	Neg	Neg	Neg	Neg	Neg	Neg
127		Conf	−	−	640	Neg	Neg	Neg	Neg	Neg	14
118		Conf	−	−	640	Neg	Neg	Neg	14	Neg	Neg
89	3	Conf	−	−^b^	160	Neg	Neg	Neg	Neg	Neg	Neg
97		Conf	−	−^b^	640	Neg	Neg	Neg	Neg	Neg	Neg
94	3	Conf	−	−^b^	640	Neg	Neg	Neg	Neg	Neg	Neg
110		Conf	−	−^b^	160	Neg	Neg	Neg	Neg	Neg	Neg
121		Conf	−	−^b^	160	14	Neg	Neg	Neg		
54	3	Conf		−	57	Neg	Neg	Neg	Neg	Neg	Neg
128		Conf	Eq	−	640	Neg	Neg	Neg	Neg	Neg	Neg
51	5	Conf	Eq	−	905	Neg	Neg	Neg	Neg	Neg	Neg
33		Conf	Eq	−	452	Neg	Neg	Neg	Neg	Neg	Neg
58	5	Conf	Eq	−	2560 ^c^	Neg	Neg	Neg	Neg	Neg	Neg
62	6	Conf	Eq	−	226	20	Neg	Neg	Neg	Neg	Neg
35	4	Conf	+	−	160	Neg	Neg	Neg	Neg	Neg	Neg
36		Conf	+	−	40	Neg	Neg	Neg	Neg	Neg	Neg
41	4	Conf	+	−	320	Neg	Neg	Neg	Neg	Neg	Neg
65	13	Conf	+	−	80	14	Neg	14	Neg	Neg	Neg
67	4	Conf	+	−	1280	Neg	Neg	Neg	Neg	Neg	28
68	6	Conf	+	−	2560 ^c^	Neg	Neg	Neg	Neg	Neg	Neg
69	5	Conf	+	−	452	Neg	Neg	Neg	Neg	Neg	Neg
70	6	Conf	+	−	640	Neg	Neg	Neg	Neg	Neg	Neg
71	8	Conf	+	−	640	Neg	Neg	Neg	Neg	Neg	14
74	4	Conf	+	−	226	Neg	Neg	Neg	Neg	Neg	Neg
75	14	Conf		−	905	Neg	Neg	Neg	Neg	Neg	Neg
79	5	Conf	+	−	640	Neg	Neg	Neg	Neg	Neg	Neg
80	7	Conf	+	−	226	Neg	Neg	Neg	Neg	Neg	Neg
81	6	Conf	+	−	640	Neg	Neg	Neg	Neg	Neg	Neg
82	6	Conf	+	−	905	Neg	Neg	Neg	Neg	Neg	Neg
86		Conf	+	−	2560 ^c^	Neg	Neg	Neg	Neg	Neg	20
91	7	Conf	+	−	1280	Neg	Neg	Neg	Neg	Neg	Neg
95	4	Conf	+	−	320	Neg	Neg	Neg	Neg	Neg	14
99		Conf	+	−	640	40	Neg	Neg	20	Neg	Neg
108		Conf	+	−	320	Neg	Neg	Neg	40	Neg	Neg
109		Conf	+	−	113	Neg	Neg	Neg	Neg	Neg	Neg
116		Conf	+	−	113	Neg	Neg	Neg	Neg	Neg	Neg
122		Conf	+	−	1280	Neg	Neg	Neg	Neg	Neg	Neg
125		Conf	+	−	160	Neg	Neg	14	Neg	Neg	Neg
											
31		Comp	−	−	226		Neg	Neg	Neg^a^		
32		Comp	−	−	226		Neg	Neg	Neg^a^		
45	10	Comp	−	−	80		Neg	Neg	Neg		
											
56	6	Unkn	+	−	80	Neg	40	20	Neg	Neg	Neg
77	6	Unkn	+	−	80	Neg	28	Neg	Neg	Neg	Neg
85		Unkn	+	−	320	98	160	57	20	Neg	Neg
78	4	Unkn	+	−	160	Neg	Neg	Neg	Neg	80	Neg
											
39	3	Neg	−	−	Neg	Neg	Neg	Neg	Neg	Neg	Neg
83	5	Neg	−	−	Neg	Neg	Neg	Neg	Neg	Neg	Neg
48	3	Neg	+	−	Neg	20	Neg	Neg	Neg	Neg	Neg
61	14	Neg	+	−	Neg	14	14	Neg	14	Neg	Neg
49	10	Neg	+	−	Neg	14	Neg	Neg	Neg		
120		Neg	+	−	20	40	Neg	20	14	Neg	Neg

NAb titre: NAb assessed by VNT, geometric mean calculated from duplicate results. neg: no NAb detected in duplicate samples (observation of cytopathic effect) for all serum dilutions tested (lowest = 20); NAb titre ≥40 considered as positive; D1–4: dengue virus 1–4; ZIK: Zika virus; WN: West Nile virus; class: classification for JE status according to the criteria set out in Figure 2; Conf: confirmed; Comp: compatible; Unkn: unknown; JEV IgG:= anti-JEG IgG detection by ELISA (Euroimmun); +: positive; Eq: equivocal; −: negative.

^a^Only one replicate tested or interpretable, the other samples were tested in duplicate.

^b^JEV IgG negative before depletion.

^c^Maximum dilution tested.

^d^Test not performed.

## Discussion

This pilot study included a large set of well-characterized patients recruited prospectively in clinical studies, with extensive VNT for JEV, DENV 1–4, ZIKV and WNV. We show that the implementation of IgG depletion prior to VNT performed on par with standard VNT (100% PPA, NPA and OPA) and also resulted in a significantly higher proportion, compared with standard VNT, of patients being classified. Of the patients with paired sera tested to confirm acute JEV infection, 74% (26/35) were classified without an IgG depletion step, in contrast to 100% when IgG depletion was included. Furthermore, IgG depletion improved the diagnostic confidence of patients classed as JE positive, from 7/26 (27%) confirmed as opposed to 19/26 (73%) compatible with standard VNT to 16/17 (94%) confirmed as opposed to 1/17 (6%) compatible with IgM-VNT. Depleting IgG also enabled a diagnosis of JE in 95% of patients for whom only a single sample was available, allowing for specific neutralization of the IgM remaining in the sample.

The high proportion of patients presenting with detectable anti-JEV IgG before depletion and a reduction in DENV neutralization titres after depletion strengthen the underlying premise of this study, that IgG complicates discrimination by VNT, especially in areas with high endemicity of other flaviviruses and increasing utilization of JEV vaccination.

A limitation is that there were not sufficient sample volumes available to perform standard and IgM-VNT in all samples. However, the testing was retrospectively performed on a relatively large number of very precious samples. It would be realistic in clinical practice to secure the serum volume (400 μL) needed for prospective IgM-VNT testing. This is one of the advantages of the new technique, that it relies on a single serum sample rather than paired sera or CSF. The efficiency of the IgG depletion was evaluated using anti-JEV IgG ELISA. We found that 84% of the anti-JEV IgG ELISA-positive sera became negative after IgG depletion. All samples with an anti-JEV IgG ELISA result <125 RU/mL were negative after IgG depletion, suggesting IgG depletion was probably incomplete in samples with high titres. Further optimization is required to ensure that depletion is fully effective, perhaps with alternative methods depending on the initial anti-JEV IgG result, such as the use of three rather than two IgG depletion columns.

The principle of removing IgG and the use of IgM as a biomarker for confirming acute infection is by no means novel. In 1973, Edelman and Pariyanonda^49^ reported a modified haemagglutination inhibition involving depletion of IgG by sucrose density gradient centrifugation of whole serum and 2-mercaptoethanol treatment. The improved discrimination of evidence for acute JE in patient samples gave rise to further work developing the widely used anti-JEV IgM ELISA.^50,51^ However, with evidence suggesting suboptimal performance of MAC-ELISA,^12^ the increasing use of the JEV vaccine, as well as hyperendemicity of DENV serotypes, the requirement for accurate diagnostic confirmation becomes even more pertinent. Although the performance of contemporaneous anti-DENV IgM ELISA and calculation of a JEV:DENV IgM ratio has improved specificity, the combination of VNT and IgG depletion (IgM-VNT) permits IgM detection with higher specificity than by using MAC-ELISA alone.

Calvert et al.^19^ showed that IgG depletion prior to neutralization testing considerably improved (15% before to 77% after IgG depletion) the differentiation of acute Zika from dengue viral infections. This has also been demonstrated for DENV infections.^42,43^ It is notable that as JE is predominantly a neurological infection, and the natural history of the immunological response is different to flavivirus infections presenting as acute febrile syndromes, by the time of clinical presentation, anti-JEV IgM and IgG is detectable in a larger proportion of patients. Therefore use of the IgM-VNT method for JE confirmation is a logical approach.

The humoral responses to JEV infection are directed mainly against antigenic epitopes on the viral envelope protein. There is major cross-reactivity with other endemic circulating flaviviruses and therefore it was crucial to test for all DENV serotypes,^52,53^ ZIKV^54^ and WNV^44^ where they are sympatric. Likewise, IgG depletion and seroneutralization might play a role in the diagnosis of DENV neurological infections for which there is considerable diagnostic uncertainty.

We acknowledge that a diagnostic accuracy study should ideally be performed with an a priori sample size calculation, prospectively testing consecutive patients with suspected neurological infection by the reference standard VNT to ascertain JE-positive and negative patient samples. However, we were unable to conduct this in this pilot study and flavivirus-naïve patients from France were included as an additional category of negative controls. That patients already had anti-JEV IgM detected in CSF or experienced JEV seroconversion reflects the role of VNT within reference centres. Further limitations include missing data due to limited sample volumes and that dilutions were 1/20 to 1/2560 for the sera. Ideally serum should be tested to the end point of dilution. IgM-VNT is a diagnostic test suited for reference centres and optimization will be required to adapt the technique to be high throughput, using protein G slurry and an automatized format for VNT testing of 1/20 to 1/5120. Additionally, not all the virus strains used were sourced from the countries where the samples were derived; the DENV strains isolated from Laos did not provide a sufficient cytopathic effect for the assay and neither ZIKV nor WNV have been isolated from patients in Laos.

In conclusion, measurement of anti-JEV IgG and the performance of IgM-VNT significantly improved performance and allowed the use of a single serum sample instead of paired sera for JE confirmation. This innovation holds promise for wider incorporation into testing algorithms in the reference confirmation of JE and DENV neurological infections.

## Supplementary Material

trac036_Supplemental_File

## Data Availability

The data underlying this article are available in the article and in its online supplementary material.

## Collaborators

We are grateful to all the SEAe study researchers, including Philippe Buchy, Em Bunnakea, Julien Cappelle, Mey Channa, Veronique Chevalier, Yoann Crabol, Philippe Dussart, Marc Eloit, Magali Herrant, Nguyen Hien, Chaw Su Hlaing, Jérôme Honnorat, Tran Thi Mai Hung, Tran Thi Thu Huong, Latt Latt Kyaw, Nguyen Van Lam, Denis Laurent, Marc Lecuit, Kyaw Linn, Olivier Lortholary, Aye Mya Min Aye, Philippe Perot, Sommanikhone Phangmanixay, Khounthavy Phongsavath, Phan Huu Phuc, Anne-Laurie Pinto, Patrice Piola, Bruno Rosset, Ky Santy, Heng Sothy, Arnaud Tarantola, Nguyen Thi Thu Thuy, Htay Htay Tin, Ommar Swe Tin, Pham Nhat An, Dang Duc Anh, Pascal Bonnet, Kimrong Bun, Danoy Chommanam, Viengmon Davong, Patrice Debré, Jean-François Delfraissy, Christian Devaux, Anousone Douangnouvong, Veasna Duong, Benoit Durand, Chanreaksmey Eng, Catherine Ferrant, Didier Fontenille, Lukas Hafner, Le Thanh Hai, Do Thu Huong, Marc Jouan, May July, Magali Lago, Jean-Paul Moatti, Bernadette Murgue, Khin Yi Oo, MengHeng Oum, Khansoudaphone Phakhounthong, Anh Tuan Pham, Do Quyen, Malee Seephonelee, Maud Seguy, Bountoy Sibounheunang, Kanarith Sim, Luong Minh Tan, Cho Thair, Win Thein, Phung Bich Thuy, Hervé Tissot-Dupont and Malavanh Vongsouvath.

## References

[1] World Health Organization . Japanese encephalitis vaccines: WHO position paper, February 2015–recommendations. Vaccine. 2016;34(3):302–3. 26232543 10.1016/j.vaccine.2015.07.057

[2] Heffelfinger JD , Li X, Batmunkh N et al. Japanese encephalitis surveillance and immunization – Asia and Western Pacific regions, 2016. MMWR Morb Mortal Wkly Rep. 2017;66(22):579–83. 28594790 10.15585/mmwr.mm6622a3 PMC5720240

[3] Hills SL , Walter EB, Atmar RL et al. Japanese encephalitis vaccine: recommendations of the Advisory Committee on Immunization Practices. MMWR Recomm Rep. 2019;68(2):1–33. 10.15585/mmwr.rr6802a1 PMC6659993 31518342

[4] Pearce JC , Learoyd TP, Langendorf BJ et al. Japanese encephalitis: the vectors, ecology and potential for expansion. J Travel Med. 2018;25(Suppl 1):S16–26. 29718435 10.1093/jtm/tay009

[5] Simon-Loriere E , Faye O, Prot M et al. Autochthonous Japanese encephalitis with yellow fever coinfection in Africa. N Engl J Med. 2017;376(15):1483–5. 28402771 10.1056/NEJMc1701600

[6] Kulkarni R , Sapkal GN, Kaushal H et al. Japanese encephalitis: a brief review on Indian perspectives. Open Virol J. 2018;12(1):121–30. 30288200 10.2174/1874357901812010121 PMC6142657

[7] Fang Y , Zhang Y, Zhou ZB et al. New strains of Japanese encephalitis virus circulating in Shanghai, China after a ten-year hiatus in local mosquito surveillance. Parasites Vectors. 2019;12:14. 30626442 10.1186/s13071-018-3267-9 PMC6327439

[8] Ojha JK , Samantaray K, Mohanty S. Assess the coverage rate of Japanese encephalitis vaccination and factors of non-compliance as reported by parents of selected areas of Khurdha. Eur J Mol Clin Med. 2021;7(11):5049–60.

[9] Do LP , Bui TM, Hasebe F et al. Molecular epidemiology of Japanese encephalitis in northern Vietnam, 1964–2011: genotype replacement. Virol J. 2015;12:51. 25889499 10.1186/s12985-015-0278-4 PMC4417254

[10] Bharucha T , Sengvilaipaseuth O, Vongsouvath M et al. Development of an improved RT-qPCR Assay for detection of Japanese encephalitis virus (JEV) RNA including a systematic review and comprehensive comparison with published methods. PLoS One. 2018;13(3):e0194412. 29570739 10.1371/journal.pone.0194412 PMC5865736

[11] Hills S , Dabbagh A, Jacobson J et al. Evidence and rationale for the World Health Organization recommended standards for Japanese encephalitis surveillance. BMC Infect Dis. 2009;9:214. 20038298 10.1186/1471-2334-9-214 PMC2809064

[12] Dubot-Peres A , Sengvilaipaseuth O, Chanthongthip A et al. How many patients with anti-JEV IgM in cerebrospinal fluid really have Japanese encephalitis? Lancet Infect Dis. 2015;15(12):1376–7. 26607119 10.1016/S1473-3099(15)00405-3

[13] Maeki T , Tajima S, Ikeda M et al. Analysis of cross-reactivity between flaviviruses with sera of patients with Japanese encephalitis showed the importance of neutralization tests for the diagnosis of Japanese encephalitis. J Infect Chemother. 2019;25(10):786–90. 31105002 10.1016/j.jiac.2019.04.003

[14] Hills S , Van Keulen A, Feser J et al. Persistence of IgM antibodies after vaccination with live attenuated Japanese encephalitis vaccine. Am J Trop Med Hyg. 2020;104(2):576–9. 33236716 10.4269/ajtmh.20-1132 PMC7866339

[15] Fatima T , Rais A, Khan E et al. Investigation of Japanese encephalitis virus as a cause of acute encephalitis in southern Pakistan, April 2015–January 2018. PLoS One. 2020;15(6):e0234584. 32530966 10.1371/journal.pone.0234584 PMC7292402

[16] Centers for Disease Control . Japanese encephalitis. Diagnostic testing. Available from: https://www.cdc.gov/japaneseencephalitis/healthcareproviders/healthcareproviders-diagnostic.html [accessed 18 April 2022].

[17] Robinson JS , Featherstone D, Vasanthapuram R et al. Evaluation of three commercially available Japanese encephalitis virus IgM enzyme-linked immunosorbent assays. Am J Trop Med Hyg. 2010;83(5):1146–55. 21036854 10.4269/ajtmh.2010.10-0212 PMC2963986

[18] Bharucha T , Shearer FM, Vongsouvath M et al. A need to raise the bar—a systematic review of temporal trends in diagnostics for Japanese encephalitis virus infection, and perspectives for future research. Int J Infect Dis. 2020;95:444–56. 32205287 10.1016/j.ijid.2020.03.039 PMC7294235

[19] Calvert AE , Boroughs KL, Laven J et al. Incorporation of IgG depletion in a neutralization assay facilitates differential diagnosis of Zika and dengue in secondary flavivirus infection cases. J Clin Microbiol. 2018;56(6):e00234–18. 29618505 10.1128/JCM.00234-18 PMC5971560

[20] Balakrishnan A , Thekkekare RJ, Sapkal G et al. Seroprevalence of Japanese encephalitis virus & West Nile virus in Alappuzha district, Kerala. Indian J Med Res. 2017;146(Suppl):S70–5. 29205199 10.4103/ijmr.IJMR_1638_15 PMC5735574

[21] Meyding-Lamadé U , Craemer E, Schnitzler P. Emerging and re-emerging viruses affecting the nervous system. Neurol Res Pract. 2019;1:20. 33324886 10.1186/s42466-019-0020-6 PMC7650110

[22] Amaro F , Sanchez-Seco MP, Vazquez A et al. The application and interpretation of IgG avidity and IgA ELISA tests to characterize Zika virus infections. Viruses. 2019;11(2):179. 10.3390/v11020179 PMC6409741 30791664

[23] Warnecke JM , Lattwein E, Saschenbrecker S et al. Added value of IgA antibodies against Zika virus non-structural protein 1 in the diagnosis of acute Zika virus infections. J Virol Methods. 2019;267:8–15. 30779938 10.1016/j.jviromet.2019.02.005

[24] Colonetti T , Rocha BVE, Grande AJ et al. Accuracy of immunoglobulin M and immunoglobulin A of saliva in early diagnosis of dengue: systematic review and meta-analysis. An Acad Bras Cienc. 2018;90(3):3147–54. 29947679 10.1590/0001-3765201820170989

[25] Nascimento EJM , Huleatt JW, Cordeiro MT et al. Development of antibody biomarkers of long term and recent dengue virus infections. J Virol Methods. 2018;257:62–8. 29684416 10.1016/j.jviromet.2018.04.009

[26] Rockstroh A , Moges B, Barzon L et al. Specific detection of dengue and Zika virus antibodies using envelope proteins with mutations in the conserved fusion loop. Emerg Microbes Infect. 2017;6(11):e99. 29116222 10.1038/emi.2017.87 PMC5717088

[27] Zhang B , Pinsky BA, Ananta JS et al. Diagnosis of Zika virus infection on a nanotechnology platform. Nat Med. 2017;23(5):548–50. 28263312 10.1038/nm.4302

[28] Huang CH , Chang YH, Lin CY et al. Shared IgG infection signatures vs. hemorrhage-restricted IgA clusters in human dengue: a phenotype of differential class-switch via *TGFβ1*. Front Immunol. 2017;8:1726. 29255469 10.3389/fimmu.2017.01726 PMC5723002

[29] Balmaseda A , Saborio S, Tellez Y et al. Evaluation of immunological markers in serum, filter-paper blood spots, and saliva for dengue diagnosis and epidemiological studies. J Clin Virol. 2008;43(3):287–91. 18783984 10.1016/j.jcv.2008.07.016

[30] Balmaseda A , Guzman MG, Hammond S et al. Diagnosis of dengue virus infection by detection of specific immunoglobulin M (IgM) and IgA antibodies in serum and saliva. Clin Diagn Lab Immunol. 2003;10(2):317–22. 12626461 10.1128/CDLI.10.2.317-322.2003 PMC150529

[31] Yap G , Sil BK, Ng LC. Use of saliva for early dengue diagnosis. PLoS Negl Trop Dis. 2011;5(5):e1046. 21572982 10.1371/journal.pntd.0001046 PMC3091836

[32] de Vasconcelos ZFM , Azevedo RC, Thompson N et al. Challenges for molecular and serological ZIKV infection confirmation. Childs Nerv Syst. 2018;34(1):79–84. 29110196 10.1007/s00381-017-3641-5

[33] Ronnberg B , Gustafsson A, Vapalahti O et al. Compensating for cross-reactions using avidity and computation in a suspension multiplex immunoassay for serotyping of Zika versus other flavivirus infections. Med Microbiol Immunol. 2017;206(5):383–401. 28852878 10.1007/s00430-017-0517-y PMC5599479

[34] Tsai WY , Youn HH, Tyson J et al. Use of urea wash ELISA to distinguish Zika and dengue virus infections. Emerg Infect Dis. 2018;24(7):1355–9. 29912689 10.3201/eid2407.171170 PMC6038735

[35] Shen WF , Galula JU, Chang GJ et al. Improving dengue viral antigens detection in dengue patient serum specimens using a low pH glycine buffer treatment. J Microbiol Immunol Infect. 2017;50(2):167–74. 26260863 10.1016/j.jmii.2015.05.008

[36] Balmaseda A , Stettler K, Medialdea-Carrera R et al. Antibody-based assay discriminates Zika virus infection from other flaviviruses. Proc Natl Acad Sci USA. 2017;114(31):8384–9. 28716913 10.1073/pnas.1704984114 PMC5547631

[37] Zhu T , He J, Chen W et al. Development of peptide-based chemiluminescence enzyme immunoassay (CLEIA) for diagnosis of dengue virus infection in human. Anal Biochem. 2018;556:112–8. 29966589 10.1016/j.ab.2018.06.030

[38] Lebani K , Jones ML, Watterson D et al. Isolation of serotype-specific antibodies against dengue virus non-structural protein 1 using phage display and application in a multiplexed serotyping assay. PLoS One. 2017;12(7):e0180669. 28683141 10.1371/journal.pone.0180669 PMC5500353

[39] Piyasena TBH , Setoh YX, Hobson-Peters J et al. Differential diagnosis of flavivirus infections in horses using viral envelope protein domain III antigens in enzyme-linked immunosorbent assay. Vector Borne Zoonotic Dis. 2017;17(12):825–35. 29083957 10.1089/vbz.2017.2172

[40] Kim DTH , Bao DT, Park H et al. Development of a novel peptide aptamer-based immunoassay to detect Zika virus in serum and urine. Theranostics. 2018;8(13):3629–42. 30026871 10.7150/thno.25955 PMC6037026

[41] Frietze KM , Pascale JM, Moreno B et al. Pathogen-specific deep sequence-coupled biopanning: a method for surveying human antibody responses. PLoS One. 2017;12(2):e0171511. 28152075 10.1371/journal.pone.0171511 PMC5289605

[42] Tsai W-Y , Durbin A, Tsai J-J et al. Complexity of neutralizing antibodies against multiple dengue virus serotypes after heterotypic immunization and secondary infection revealed by in-depth analysis of cross-reactive antibodies. J Virol. 2015;89(14):7348–62. 25972550 10.1128/JVI.00273-15 PMC4473561

[43] Zainal N , Tan KK, Johari J et al. Sera of patients with systemic lupus erythematosus cross-neutralizes dengue viruses. Microbiol Immunol. 2018;62(10):659–72. 30259549 10.1111/1348-0421.12652

[44] Dubot-Peres A , Mayxay M, Phetsouvanh R et al. Management of central nervous system infections, Vientiane, Laos, 2003–2011. Emerg Infect Dis. 2019;25(5):898–910. 31002063 10.3201/eid2505.180914 PMC6478220

[45] Pommier JD , Gorman C, Crabol J et al. on behalf of the SEAe Consortium. An extensive three-year investigation of childhood encephalitis in the Greater Mekong region - The South East Asia encephalitis project. Lancet Global Health (in press).

[46] Cachay R , Schwalb A, Acevedo-Rodriguez JG et al. Zika virus seroprevalence in two districts of Chincha, Ica, Peru: a cross-sectional study. Am J Trop Med Hyg. 2021;106(1):192–8. 34814106 10.4269/ajtmh.20-1339 PMC8733524

[47] Nurtop E , Villarroel PMS, Pastorino B et al. Combination of ELISA screening and seroneutralisation tests to expedite Zika virus seroprevalence studies. Virology J. 2018;15(1):192. 30587193 10.1186/s12985-018-1105-5 PMC6307276

[48] Sakhria S , Bichaud L, Mensi M et al. Co-circulation of Toscana virus and Punique virus in northern Tunisia: a microneutralisation-based seroprevalence study. PLoS Negl Trop Dis. 2013;7(9):e2429. 24069484 10.1371/journal.pntd.0002429 PMC3772032

[49] Edelman R , Pariyanonda A. Human immunoglobulin M antibody in the sero-diagnosis of Japanese encephalitis virus infections. Am J Epidemiol. 1973;98(1):29–38. 4360765 10.1093/oxfordjournals.aje.a121529

[50] Burke DS , Nisalak A, Ussery MA. Antibody capture immunoassay detection of Japanese encephalitis virus immunoglobulin M and G antibodies in cerebrospinal fluid. J Clin Microbiol. 1982;16(6):1034–42. 7161371 10.1128/jcm.16.6.1034-1042.1982 PMC272535

[51] Burke DS , Nisalak A. Detection of Japanese encephalitis virus immunoglobulin M antibodies in serum by antibody capture radioimmunoassay. J Clin Microbiol. 1982;15(3):353–61. 6281305 10.1128/jcm.15.3.353-361.1982 PMC272099

[52] Castonguay-Vanier J , Klitting R, Sengvilaipaseuth O et al. Molecular epidemiology of dengue viruses in three provinces of Lao PDR, 2006–2010. PLoS Negl Trop Dis. 2018;12(1):e0006203. 29377886 10.1371/journal.pntd.0006203 PMC5805359

[53] Mayxay M , Castonguay-Vanier J, Chansamouth V et al. Causes of non-malarial fever in Laos: a prospective study. Lancet Glob Health. 2013;1(1):e46–54. 24748368 10.1016/S2214-109X(13)70008-1 PMC3986032

[54] Pastorino B , Sengvilaipaseuth O, Chanthongthip A et al. Low Zika virus seroprevalence in Vientiane, Laos, 2003–2015. Am J Trop Med Hyg. 2019;100(3):639–42. 30693859 10.4269/ajtmh.18-0439 PMC6402904

