# Supplementary material for: Immunoglobulin M seroneutralization for improved confirmation of Japanese encephalitis virus infection in a flavivirus-endemic area
Source: Trans R Soc Trop Med Hyg. 2022 May 18;116(11):1032–42. doi: 10.1093/trstmh/trac036 (PMC9623734; doi:10.1093/trstmh/trac036)
Supplement: trac036_Supplemental_File [file trac036_supplemental_file.zip › S2 Data.docx]

**S2 Data:** **Samples for which there was insufficient sera to perform JEV IgG ELISA after IgG depletion and/or IgM VNT**

Table S2A : Virus neutralisation test antibody titer in acute and follow-up serum samples for patients with positive anti-JEV IgM capture ELISA

| **Patient number** | **Sample type** | **Days of Illness** | **Before IgG depletion = Standard VNT** | | | | | | | | | **After IgG depletion = IgM VNT** | | | | | | | | |
| --- | --- | --- | --- | --- | --- | --- | --- | --- | --- | --- | --- | --- | --- | --- | --- | --- | --- | --- | --- | --- |
|  |  |  | **Class** | **JEV IgG** | **NAb titer** | | | | | | | **Class** | **JEV IgG** | **NAb titer** | | | | | | |
|  |  |  |  |  | **JEV** | **D1** | **D2** | **D3** | **D4** | **ZIK** | **WN** |  |  | **JEV** | **D1** | **D2** | **D3** | **D4** | **ZIK** | **WN** |
| 1615 | Adm | 5 | Comp | + | 2560 | 28 | neg | neg | 14 |  |  |  |  | 1280* | neg* | neg* | neg* |  | neg* |  |
|  | FU | 28 |  | + | 2560 | 80 | 20 | neg | 28 |  |  |  |  | 2560* | neg* | neg* | neg* | neg* | neg* | 40* |
| 1493 | Adm | 5 | Comp |  | 320 | neg | neg |  | neg |  |  |  |  | 1280 | neg | neg | neg |  |  |  |
|  | FU | 41 |  | + | 640 | neg | neg |  | 20 |  |  |  |  | 320 | neg | neg | neg | neg | neg | neg |
| 923 | Adm | 7 | Comp | + | 80 | 40 | 640 |  | neg |  |  |  |  | 640 | neg | neg | neg |  |  |  |
|  | FU | 27 |  | + | 640 | 40 | 160 |  | 160 |  |  |  |  | 640 | neg | neg | neg |  |  |  |
| 1078 | Adm | 8 | Comp | - | 640 | neg | neg | neg |  |  |  |  |  |  |  |  |  |  |  |  |
|  | FU | 20 |  | + | 1280 |  | neg* | neg |  |  |  |  |  |  |  |  |  |  |  |  |
| 1093 | Adm | 1 | Comp | + | 905 | 28 | neg | neg | neg |  |  |  |  |  |  |  |  |  |  |  |
|  | FU | 22 |  | + | 1280 |  | neg* | neg |  |  |  |  |  |  |  |  |  |  |  |  |
| 1297 | Adm | 6 | Comp | - | 1280 |  |  | neg* |  |  |  |  |  |  |  |  |  |  |  |  |
|  | FU | 18 |  | + | 1280 |  | neg* | neg |  |  |  |  |  |  |  |  |  |  |  |  |
| 1330 | Adm | 6 | Comp | + | 1280 |  | neg | neg | neg |  |  |  |  |  |  |  |  |  |  |  |
|  | FU | 18 |  | + | 1280 |  | neg* | neg |  |  |  |  |  |  |  |  |  |  |  |  |
| 545 | Adm | 25 | Comp |  | 113 | neg | neg |  | neg |  |  |  |  |  |  |  |  |  |  |  |
|  | FU | 69 |  |  | 640 | neg | neg |  | neg |  |  |  |  |  |  |  |  |  |  |  |
| 933 | Adm | 5 | Comp |  | 320 | neg | neg |  | neg |  |  |  |  |  |  |  |  |  |  |  |
|  | FU | 10 |  |  | 640 | neg | neg |  | neg |  |  |  |  |  |  |  |  |  |  |  |
| 1433 | Adm | 7 | Comp |  | 80 | neg | neg |  | neg |  |  |  |  |  |  |  |  |  |  |  |
|  | FU | 13 |  |  | 640 | neg | neg |  | neg |  |  |  |  |  |  |  |  |  |  |  |
| 1468 | Adm | 4 | Comp |  | 160 | neg | neg |  | neg |  |  |  |  |  |  |  |  |  |  |  |
|  | FU | 28 |  |  | 640 | neg | neg |  | neg |  |  |  |  |  |  |  |  |  |  |  |
| 930 | Adm | 13 | Unkn |  | 160 | neg | neg |  | 40 |  |  |  |  | 1280 | neg | neg | neg |  |  |  |
|  | FU | 24 |  | + | ** | neg | neg |  | neg |  |  |  |  | 320 | neg | neg | neg | neg | neg | neg |
| 1979 | Adm | 1 | Unkn | + | 1280 | 20 | 320 | 453 | 160 |  |  |  | - | 226 |  |  |  |  |  |  |
|  | FU | 38 |  | + | 2560 | 80 | 640 | 320 | 640 |  |  |  | - |  |  |  |  |  |  |  |
| 1604 | Adm | 4 | Unkn | + | 1810 | 2560 | 2560 | 2560 | 2560 |  |  |  |  | 640* | 40* | 80* | 40* | neg* | neg* |  |
|  | FU | 53 |  | + | 2560 | 2560 | 2560 | 2560 | 2560 |  |  |  |  | 1280 | 640 | 80 | 320 | neg | 20 |  |
| 939 | Adm | 5 | Unkn | + | 160 | neg | 160 |  |  |  |  |  |  | 640 | neg | neg | neg |  |  |  |
|  | FU | 14 |  | + | 320 | 80 | 80 |  | 640 |  |  |  |  | 640 | neg | neg | neg |  |  |  |
| 793 | Adm | 7 | Unkn^+^ | - | 320 | neg | neg |  | neg |  |  |  |  |  |  |  |  |  |  |  |
|  | FU | 18 |  | + | neg | neg | 320 |  | 640 |  |  |  |  | neg | neg | neg | neg |  |  |  |
| 7061 | Adm | 13 | Unkn | + | 905 | 640 | 640* | 2560 | 640 | 57 | 320 |  |  |  |  |  |  |  |  |  |
|  | FU | 33 |  | + | 2560 | 905 | 1810 | 2560 | 640 | 57 | 160 |  |  |  |  |  |  |  |  |  |

Adm= serum on admission; FU=serum at follow-up; NAb titer=Neutralising antibody titer assessed by virus neutralization test (VNT), geometric mean calculated from duplicate results, **=indeterminate, NAb titer underlined to indicate the maximum dilution tested, neg=no NAb detected in duplicate samples (observation of cytopathic effect) for all serum dilutions tested (lowest one=20); NAb titer ≥40 considered as positive; JEV=Japanese encephalitis virus; D1-4=Dengue virus 1-4; ZIK=Zika virus; WN=West Nile virus; Class=classification for JE status according to criteria in Table 2; Conf=Confirmed; Comp=Compatible; Ukn=Unknown; JEV IgG=anti-JEG IgG detection by ELISA (Euroimmun); +=Positive; eq=Equivocal; -=Negative. *Only one replicate tested or interpretable, the other samples were tested in duplicate. ^+^ This sample would be classified as confirmed JE based on a single sample, however as the FU sample is negative it is more logically classified as Unkn.

Table S2B: Virus neutralisation test antibody titer for patients with only a single acute serum sample

| **Patient number** | **Days of Illness** |  | **Before IgG depletion** | **After IgG depletion = IgM VNT** | | | | | | | |
| --- | --- | --- | --- | --- | --- | --- | --- | --- | --- | --- | --- |
|  |  | **Class** | **JEV IgG** | **JEV IgG** | **NAb titer** | | | | | | |
|  |  |  |  |  | **JEV** | **D1** | **D2** | **D3** | **D4** | **ZIK** | **WN** |
| 42 | 3 |  | + |  | 640 | neg | neg | neg | neg | neg |  |
| 72 | 5 |  | + |  | 40 | 14 | neg | neg | neg | neg | neg |
| 90 | 3 |  | + |  | 160 | 20 | neg | neg | neg |  |  |
| 93 | 1 |  | + |  | 1280 |  | neg | neg | neg | neg |  |
| 96 | 9 |  | + |  | 1280 |  | neg | neg | neg |  |  |
| 100 |  |  | + |  | 28 |  |  | neg | neg |  |  |
| 107 |  |  | + |  | 2560 |  | neg | neg | neg | neg |  |
| 117 |  |  | + | eq | 1280 | 80 | neg | neg | 28 | neg | neg |
| 119 |  |  | + | eq | 98 | 14 | neg | neg | neg | neg | neg |
| 113 |  |  | + | + | 320 | 57 | neg | 80 | 40 | neg | neg |
| 114 |  |  | + | + | 640 | 28 | neg | 20 | 14 | neg | neg |
| 43 | 14 |  | + | + | 1280 | neg | neg | neg | neg | neg | neg |
| 84 |  |  | + | + | 640 | neg | neg | neg | neg | neg | neg |
| 115 |  |  | + | + | 452 | neg | neg | neg | neg | neg | neg |
| 124 |  |  | + | + | 320 | neg | neg | neg | neg | neg | neg |
| 126 |  |  | + | + | 452 | 40 | neg | 80 | neg | neg | neg |
| 50 | 4 |  | + |  | 40 | 80 | 40 | 80 | neg | neg | neg |
| 55 | 6 |  | + | eq | 80 | 160 | 40 | neg | neg | neg | neg |
| 46 | 4 |  | + | + | 320 | 226 | 40 | 40 | neg | neg | neg |
| 63 | 6 |  | + | + | 320 | 226 | 160 | 113 | neg | 57 | 28 |
| 106 |  |  | + | + | 320 | 160 | neg | 40 | 28 | neg | neg |
| 123 |  |  | + | + | 80 | 905 | 40 | 1280 | 320 | neg | 14 |

NAb titer=Neutralising antibody titer assessed by virus neutralization test (VNT), geometric mean calculated from duplicate results, NAb titer underlined to indicate the maximum dilution tested; neg=no NAb detected in duplicate samples (observation of cytopathic effect) for all serum dilutions tested (lowest one=20); NAb titer ≥40 considered as positive;JEV=Japanese encephalitis virus; D1-4=Dengue virus 1-4; ZIK=Zika virus; WN=West Nile virus; Class=classification for JE status according to the criteria set out in Table 2; Conf=Confirmed; Comp=Compatible; Ukn=Unknown; JEV IgG=anti-JEG IgG detection by ELISA (Euroimmun); +=Positive; eq=Equivocal; -=Negative; *Only one replicate tested or interpretable, the other samples were tested in duplicate. Grey shading=test not performed.
