# Supplementary material for: Immunoglobulin M seroneutralization for improved confirmation of Japanese encephalitis virus infection in a flavivirus-endemic area
Source: Trans R Soc Trop Med Hyg. 2022 May 18;116(11):1032–42. doi: 10.1093/trstmh/trac036 (PMC9623734; doi:10.1093/trstmh/trac036)
Supplement: trac036_Supplemental_File [file trac036_supplemental_file.zip › S3 Data.docx]

S3 Table: JEV neutralisation test antibody titre for negative controls

|  |  |  | **Before IgG depletion** | | **After IgG depletion** | |
| --- | --- | --- | --- | --- | --- | --- |
| **Patient number** | **Category** | **Sample type** | **JEV IgG** | **JEV NAb titre** | **JEV IgG** | **JEV NAb titre** |
|  |  |  |  |  |  |  |
| Sera_M00212_A2 | Healthy flavivirus naïve serum from France | Blood donor | - | neg | - | neg |
| Sera_M00212_A3 | Healthy flavivirus naïve serum from France | Blood donor | - | neg | - | neg |
| Sera_M00212_A5 | Healthy flavivirus naïve serum from France | Blood donor | - | neg | - | neg |
| Sera_M00212_A9 | Healthy flavivirus naïve serum from France | Blood donor | - | neg | - | neg |
| Sera_M00212_A11 | Healthy flavivirus naïve serum from France | Blood donor | - | neg | - | neg |
| Sera_M00212_B1 | Healthy flavivirus naïve serum from France | Blood donor | - | neg | - | neg |
| Sera_M00212_B2 | Healthy flavivirus naïve serum from France | Blood donor | - | neg | - | neg |
| Sera_M00212_B3 | Healthy flavivirus naïve serum from France | Blood donor | eq | neg | - | neg |
| Sera_M00212_B4 | Healthy flavivirus naïve serum from France | Blood donor | - | neg | - | neg |
| Sera_M00212_B9 | Healthy flavivirus naïve serum from France | Blood donor | - | neg | - | neg |
|  |  |  |  |  |  |  |
| ZIKA_2 | Zika convalescent serum from South America | FU | + | neg | - | neg |
| ZIKA_23 | Zika convalescent serum from South America | FU | - | neg | - | neg |
| ZIKA_95 | Zika convalescent serum from South America | FU | + | neg | - | neg |
| ZIKA_149 | Zika convalescent serum from South America | FU | + | neg | - | neg |
|  |  |  |  |  |  |  |
| 1443 | Dengue acute serum from Laos CNS study | Adm | - | 20 | - | neg |
|  |  | FU | - | 28 | - | neg |
| 869 | Dengue acute serum from Laos CNS study | Adm | - | neg | - | neg |
|  |  | FU | - | neg | - | neg |
| 1103 | Dengue acute serum from Laos CNS study | Adm | - | 160 | - | neg |
|  |  | FU | + | 905 | - | neg |
| 949 | Dengue acute serum from Laos CNS study | Adm | + | 160 | - | neg |
|  |  | FU | + | 113 | - | neg |
| 951 | Dengue acute serum from Laos CNS study | Adm | + | neg | - | neg |
|  |  | FU | + | 453 | - | neg |
| 1301 | Dengue acute serum from Laos CNS study | Adm | + | 57 | - | neg |
|  |  | FU | + | 160 | - | neg |
| L170 | Dengue acute serum from Laos CNS study | Adm | + | 160 | - | neg |
|  |  | FU | + | 226 | - | neg |
| 953 | Dengue acute serum from Laos CNS study | Adm | + | 113 | - | neg |
|  |  | FU | + | 80 | - | neg |
| L37 | Dengue acute serum from Laos CNS study | Adm | + | 160 | - | neg |
|  |  | FU | + | 320 | - | neg |
| L90 | Dengue acute serum from Laos CNS study | Adm | + | 453 | - | neg |
|  |  | FU | + | 905 | - | neg |
| 790 | Dengue acute serum from Laos CNS study | Adm | + | 453 | - | 28 |
|  |  | FU | + | 1280 | - | 40 |
| L221 | Dengue acute serum from Laos CNS study | Adm | + | neg | - | ** |
|  |  | FU | + | 226 | - | ** |

Adm= serum on admission; FU=serum at follow-up; NAb titer=Neutralising antibody titer assessed by virus neutralization test (VNT), geometric mean calculated from duplicate results, NAb titer underlined to indicate the maximum dilution tested, neg=no NAb detected (observation of cytopathic effect) for all serum dilutions tested (lowest one=20), NAb titer ≥40 considered as positive; JEV=Japanese encephalitis virus; JEV IgG=anti-JEG IgG detection by ELISA (Euroimmun); +=Positive; eq=Equivocal; -=Negative; ** Neither replicate tested or interpretable.

Samples with no detection of anti-JEV antibody are highlighted in green, and the ones with anti-JEV antibodies detected (either by ELISA or VNT) are highlighted in blue.
