# Supplementary figures and images for: Immunoglobulin M seroneutralization for improved confirmation of Japanese encephalitis virus infection in a flavivirus-endemic area
Source: Trans R Soc Trop Med Hyg. 2022 May 18;116(11):1032–42. doi: 10.1093/trstmh/trac036 (PMC9623734; doi:10.1093/trstmh/trac036)

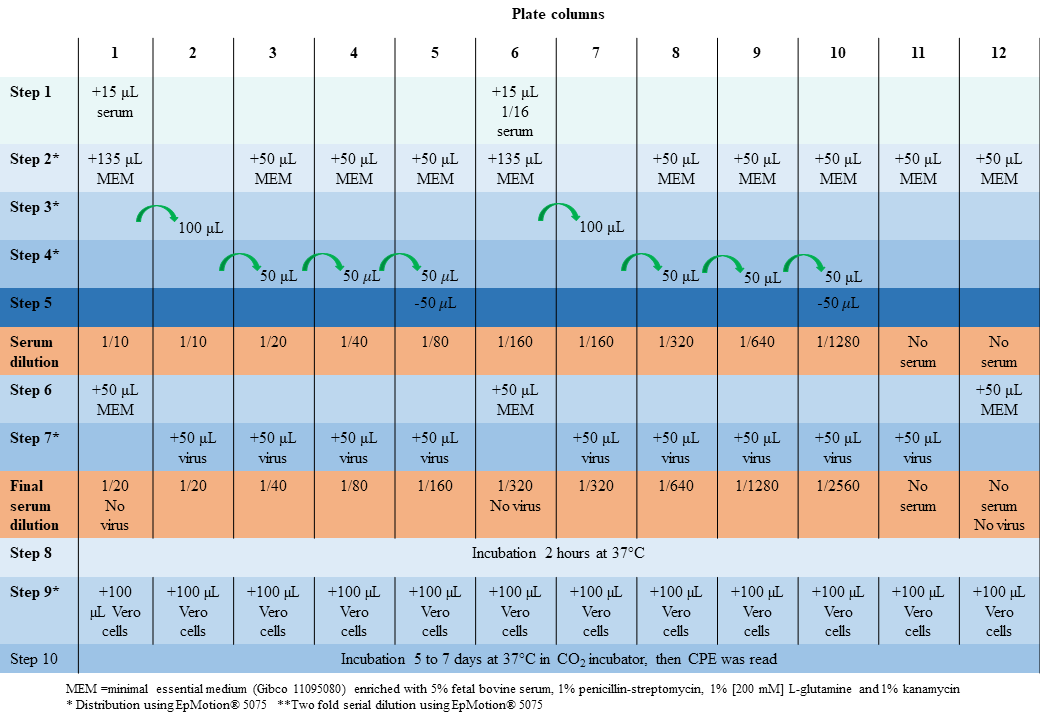

Supplement: trac036_Supplemental_File [file trac036_supplemental_file.zip › S1_Figure.png]
